# Persistent nodal disease with squamous differentiation and spatial immune heterogeneity after chemoimmunotherapy in biliary tract cancer: a high-resolution pathological analysis

**DOI:** 10.3389/fimmu.2026.1855354

**Published:** 2026-10-23

**Authors:** Hideki Sasanuma, Keita Suto, Yuki Kimura, Yuichi Aoki, Masanobu Taguchi, Naoya Kasahara, Kazue Morishima, Hideyuki Ohzawa, Naoki Sano, Noriyoshi Fukushima, Hironori Yamaguchi, Naohiro Sata

**Affiliations:** 1 Division of Gastroenterological, General and Transplant Surgery, Department of Surgery, Jichi Medical University, Shimotsuke, Tochigi, Japan; 2 Department of Clinical Oncology, Jichi Medical University Hospital, Shimotsuke, Tochigi, Japan; 3 Department of Diagnostic Pathology, Jichi Medical University Hospital, Shimotsuke, Tochigi, Japan

**Keywords:** biliary tract cancer, chemoimmunotherapy, lymph node metastasis, spatial immune profiling, spatial pathology, squamous differentiation

## Abstract

**Background:**

Heterogeneous responses across disease sites may occur after systemic therapy for biliary tract cancer (BTC), but their pathological and immune features remain poorly characterized. We report a case with pathological complete response (pCR) at the primary and pulmonary metastatic sites but persistent nodal disease after sequential chemotherapy and immunotherapy.

**Case presentation:**

A man in his 50s with metastatic perihilar cholangiocarcinoma received gemcitabine plus cisplatin. Cisplatin was discontinued because of an allergic reaction without radiographic progression, followed by gemcitabine plus durvalumab, gemcitabine alone, and durvalumab maintenance. Resection demonstrated pCR in the primary biliary lesion and a pulmonary metastasis, whereas viable carcinoma persisted in 3 of 22 regional lymph nodes. All three metastatic lymph nodes showed prominent keratinizing squamous differentiation with p40 expression and loss of CK7 expression. Spatial analysis of one lymph node showed higher CD8-positive cell abundance at the tumor margin than in the core by both positive-cell proportion and cell density. FoxP3-positive cells also showed a margin-predominant distribution, whereas LAG-3 expression and PD-L1 H-score were higher in the core and TIM-3 was broadly distributed. Another lymph node showed a different CD8 distribution, indicating inter-nodal heterogeneity.

**Conclusion:**

This case shows site-specific pathological heterogeneity after chemoimmunotherapy for BTC, with persistent nodal disease despite pCR at the primary and pulmonary metastatic sites. Residual nodal metastases showed keratinizing squamous differentiation and inter-nodal heterogeneity in immune-cell distribution. Because paired pretreatment nodal tissue, molecular or clonality analyses, cell type-specific checkpoint assessment, and spatial immune profiling were unavailable, the biological basis and generalizability remain unresolved.

## 1 Introduction

Chemoimmunotherapy can produce substantial antitumor effects in advanced biliary tract cancer (BTC) (1). However, treatment responses may differ across anatomical sites (2), and spatial immune heterogeneity has been demonstrated in cholangiocarcinoma (3). In some patients, marked regression is observed in the primary lesion and selected metastatic deposits, whereas viable tumor cells persist at other sites despite the same systemic exposure (2). Such discordant responses suggest that local tumor and immune microenvironments may contribute to site-specific differences in treatment response (4).

Several non-mutually exclusive mechanisms have been proposed to contribute to site-specific resistance (4, 5). Immune exclusion, characterized by the accumulation of cytotoxic T cells in stromal regions with limited penetration into tumor nests, has been described across multiple tumor types (5). Lineage plasticity, in which tumor cells acquire altered phenotypic states during tumor evolution or treatment, has also been associated with therapeutic resistance (6). However, the spatial and pathological features associated with heterogeneous treatment responses remain incompletely characterized in BTC (7–10), particularly after combined chemotherapy and immune checkpoint blockade.

Regional lymph nodes are relevant sites for examining such heterogeneity because they serve both as sites of metastatic spread and as organized immune microenvironments in which tumor–host interactions may differ from those at the primary tumor or distant metastatic sites (11). Persistent nodal disease despite a favorable response at other disease sites may therefore provide an opportunity to characterize pathological and spatial immune differences associated with site-specific treatment response.

Here, we report a high-resolution pathological analysis of BTC with discordant pathological responses after chemoimmunotherapy. The primary biliary lesion and a pulmonary metastasis showed pathological complete response, whereas viable carcinoma persisted in regional lymph nodes. We characterized the morphology and immunophenotype of the residual nodal metastases and performed detailed spatial immune profiling in one metastatic lymph node, supplemented by additional immune assessment of another nodal metastasis. This case provides a descriptive, hypothesis-generating example of persistent nodal disease with squamous differentiation and inter-nodal immune heterogeneity after chemoimmunotherapy.

## 2 Materials and methods

### 2.1 Patient, treatment, and specimen collection

This study was approved by the Institutional Review Board of Jichi Medical University (Approval No. A21-064) and was conducted in accordance with the Declaration of Helsinki. Written informed consent was obtained from the patient.

We analyzed surgical specimens from a patient with metastatic perihilar cholangiocarcinoma who received sequential systemic therapy followed by surgical resection. The analyzed specimens included a pulmonary metastasis resected by video-assisted thoracic surgery (VATS), the primary biliary lesion, and 22 regional lymph nodes collected during pylorus-preserving pancreatoduodenectomy (PPPD). Systemic therapy was initiated with gemcitabine plus cisplatin. After nine cycles, cisplatin was discontinued because of an allergic reaction, 5.5 months after treatment initiation. Imaging at that time showed stable disease without radiographic progression. The patient subsequently received one cycle of gemcitabine plus durvalumab, followed by one cycle of gemcitabine alone and 6 months of durvalumab maintenance monotherapy.

### 2.2 Radiologic and pathological response assessment

Tumor response was evaluated by contrast-enhanced computed tomography and magnetic resonance imaging every 8–12 weeks. Radiographic response was assessed according to the Response Evaluation Criteria in Solid Tumors (RECIST) version 1.1 (12).

Pathological treatment response in the resected specimens was assessed histologically based on the presence and extent of residual viable tumor cells. The Evans grading system was additionally applied as a standardized descriptive histopathological framework in this case rather than as a disease-specific validated grading system for biliary tract cancer (13). Evans grade IV indicates the complete absence of viable tumor cells (13).

### 2.3 Histopathology and immunohistochemistry

Resected tissues were fixed in 10% neutral-buffered formalin for 24–48 hours and embedded in paraffin. Serial 4-μm sections were prepared for hematoxylin and eosin staining and immunohistochemical analysis. Immunohistochemical staining was performed using an automated platform (Bond-Max, Leica Biosystems, Melbourne, Australia).

For lineage characterization, antibodies against p40 (ΔNp63) and cytokeratin 7 (CK7) were used. To characterize the immune microenvironment, staining was performed for CD8, FoxP3, PD-L1, LAG-3, and TIM-3. Additional staining for carcinoembryonic antigen (CEA) and CD68 was performed in one metastatic lymph node to further characterize histologically heterogeneous areas. Appropriate positive and negative controls were included in all staining runs. Staining conditions were optimized to achieve reproducible signal detection suitable for digital image quantification.

### 2.4 Whole-slide imaging and digital spatial immune analysis

Histological slides were digitized at ×40 magnification using a high-resolution whole-slide scanner. Digital image analysis was performed using QuPath software (version 0.6.0) (14). Tumor regions were initially identified by automated tissue detection and then refined by manual annotation. All tumor annotations were reviewed by a board-certified pathologist. Cells within the annotated tumor regions were identified and classified using the digital cell analysis functions in QuPath.

For spatial immune analysis, two tumor compartments were defined using predefined morphological criteria. The tumor margin was operationally defined as the region within 50 μm of the tumor–stroma interface to standardize spatial segmentation across the analyzed regions. This threshold was used for spatial comparison and was not intended to represent a biologically validated boundary of immune exclusion. The tumor core was defined as internal viable tumor nests, including keratinized squamous areas. These compartments were defined relative to the tumor–stroma interface and viable tumor morphology rather than according to normal lymph-node anatomical compartments such as the cortex, paracortex, or medulla.

Detailed quantitative spatial analysis was performed in one metastatic lymph node. This lymph node was selected because it showed prominent keratinizing squamous differentiation and preserved tissue architecture that allowed reproducible delineation of the tumor–stroma interface and internal tumor nests. Quantitative analysis was performed within the entire manually annotated tumor margin and tumor core regions using QuPath. All detected cells were defined as nucleated cells automatically identified by the QuPath cell detection algorithm using the hematoxylin optical density channel within the annotated regions. Positive cells were classified according to marker-specific 3,3′-diaminobenzidine (DAB) optical density thresholds. Cell detection and positive-cell classification were visually reviewed by a board-certified pathologist to confirm concordance with the corresponding immunohistochemical staining patterns.

CD8-positive and FoxP3-positive cells were quantified as the percentage of positive cells among all detected cells. For CD8, positive-cell density (positive cells/mm²) was additionally calculated to assess the potential influence of differences in total cellular composition between compartments. PD-L1, LAG-3, and TIM-3 expression was quantified as the proportion of positive cells. These analyses did not distinguish marker expression according to specific tumor or immune cell populations. PD-L1 expression was additionally evaluated using the H-score (range, 0–300), calculated as follows: H-score = 1 × percentage of weakly positive cells + 2 × percentage of moderately positive cells + 3 × percentage of strongly positive cells. Separate tumor proportion score (TPS) and immune cell (IC) scoring were not performed because the analysis was designed to compare the spatial distribution of PD-L1 staining between the tumor margin and tumor core rather than cell type-specific PD-L1 expression.

In another metastatic lymph node, additional CD8 and PD-L1 staining was evaluated descriptively by histological review to characterize the spatial distribution of these markers in a separate nodal metastasis. This additional evaluation was not included in the quantitative margin-versus-core analysis described above.

Representative high-power images shown in the figures were selected only to illustrate the corresponding annotated regions and were not used for quantitative measurements. The annotated areas and total analyzed cell numbers for each immunohistochemical marker included in the quantitative spatial analysis are summarized in **Supplementary Table S1**. Detailed QuPath cell detection and marker classification parameters are provided in **Supplementary Table S2** to facilitate reproducibility.

### 2.5 Statistical analysis

For statistical comparisons, marker-specific quantitative values were calculated separately for five analyzed regions within each tumor compartment. Positive-cell proportions were used for CD8, FoxP3, LAG-3, and TIM-3, whereas H-scores were used for PD-L1. For CD8, positive-cell density (positive cells/mm²) was additionally calculated and compared between compartments to assess the potential influence of differences in cellular composition on the positive-cell proportion.

The distributions of these regional values were compared between the tumor margin and tumor core using the Wilcoxon rank-sum test. All statistical analyses were performed using JMP version 18.2.2 (SAS Institute Inc., Cary, NC, USA). All tests were two-sided. Because the five regional measurements within each compartment were derived from a single metastatic lymph node and did not represent independent biological replicates, P values were interpreted descriptively in this exploratory within-lesion analysis and were not used for population-level inference.

## 3 Results

### 3.1 Discordant pathological response across disease sites after sequential systemic therapy

A man in his 50s was diagnosed with perihilar adenocarcinoma based on biliary cytology and biopsy. He received nine cycles of gemcitabine plus cisplatin. At 5.5 months after treatment initiation, cisplatin was discontinued because of an allergic reaction. Imaging at that time showed stable disease without radiographic progression. He subsequently received one cycle of gemcitabine plus durvalumab, followed by one cycle of gemcitabine alone and 6 months of durvalumab maintenance monotherapy.

At 12 months, the pulmonary lesions remained radiographically stable. However, video-assisted thoracic surgery (VATS) of a representative pulmonary nodule demonstrated fibrotic scar tissue without viable tumor cells, confirming pathological complete response (pCR). Biliary biopsies obtained after systemic therapy showed only atypical cells. On the basis of these findings, radical surgery was performed at 16 months. Pylorus-preserving pancreatoduodenectomy (PPPD) revealed marked fibrosis and sclerosis at the primary site without viable tumor cells. The patient subsequently received adjuvant S-1 therapy and remains disease-free 37 months after treatment initiation.

These findings showed a discordant pathological response pattern, with pathological clearance at the primary and pulmonary metastatic sites despite persistent regional nodal disease (**Figure 1**).

**Figure 1:**
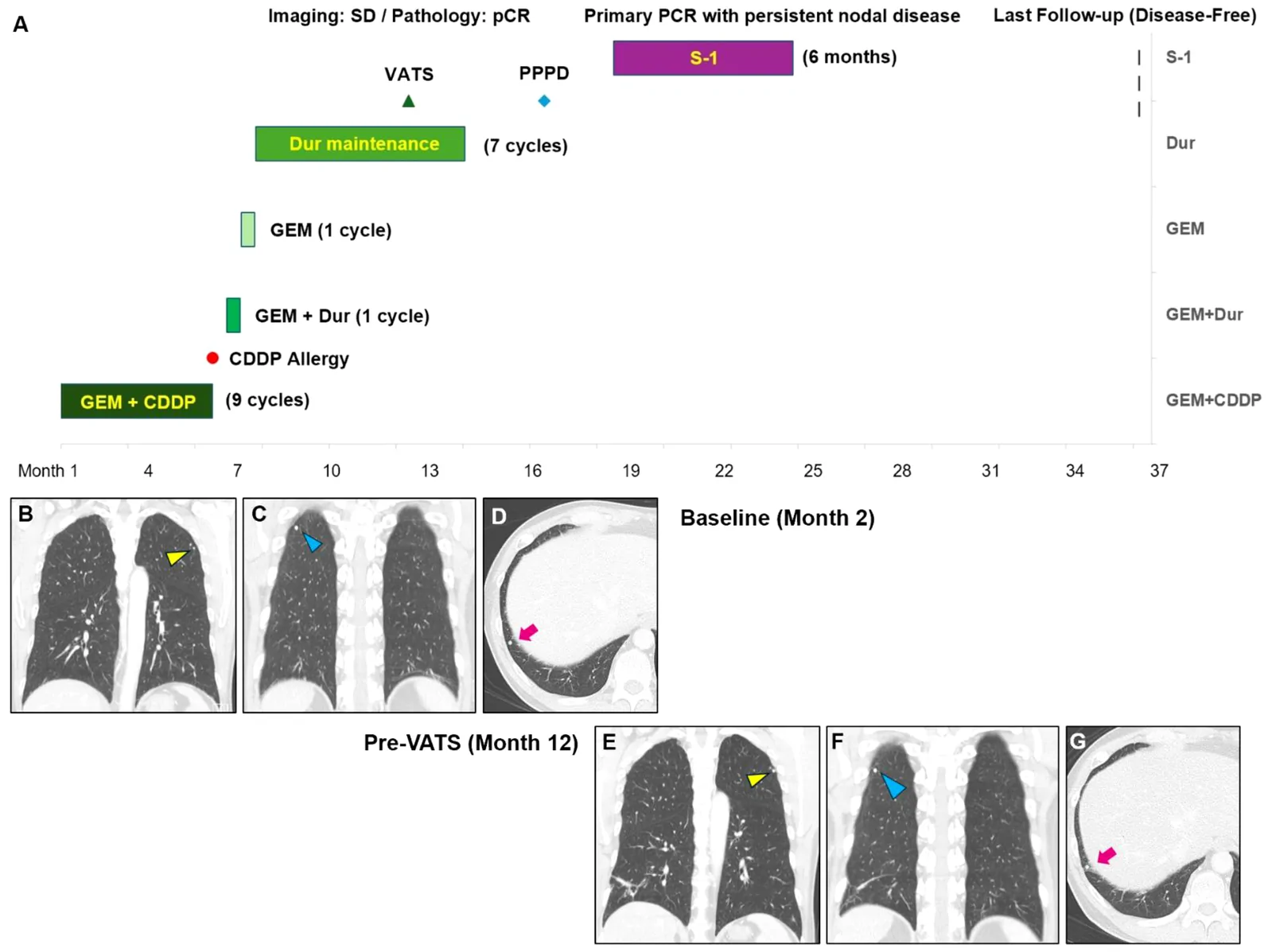
Discordant pathological response across disease sites after sequential chemoimmunotherapy. **(A)** Clinical timeline showing sequential systemic therapy, radiographic evaluation, surgical interventions, and clinical outcome. The patient initially received nine cycles of gemcitabine plus cisplatin (GC). After a cisplatin-related allergic reaction (red circle), cisplatin was discontinued, and treatment was transitioned to one cycle of gemcitabine plus durvalumab, followed by one cycle of gemcitabine alone and seven cycles of durvalumab maintenance monotherapy over 6 months. This treatment modification was prompted by the allergic reaction rather than radiographic disease progression. Radiographic assessment at Month 10 demonstrated stable disease (SD) in the pulmonary nodules. Video-assisted thoracic surgery (VATS; green triangle) was performed at Month 12 to evaluate a representative pulmonary lesion, and histopathological examination demonstrated pathological complete response (pCR). Pylorus-preserving pancreatoduodenectomy (PPPD; blue diamond) was subsequently performed at Month 16. The primary biliary lesion also showed pCR, whereas viable metastatic carcinoma persisted in regional lymph nodes. Adjuvant S-1 therapy was administered for 6 months after surgery. The patient remains disease-free 37 months after treatment initiation. **(B–D)** Baseline chest computed tomography (CT) images obtained at Month 2 showing representative pulmonary nodules in the left upper lung **(B)**; yellow arrowheads), right upper lung **(C)**; cyan arrowheads), and right lower lung **(D)**; magenta arrow). **(E–G)** Corresponding preoperative CT images obtained at Month 12 after systemic therapy. The pulmonary lesion resected by VATS (magenta arrows in **(D, G)** remained radiographically stable but was pathologically confirmed as pCR, illustrating discordance between radiographic appearance and pathological response. CDDP, cisplatin; CT, computed tomography; Dur, durvalumab; GC, gemcitabine plus cisplatin; GEM, gemcitabine; pCR, pathological complete response; PPPD, pylorus-preserving pancreatoduodenectomy; S-1, tegafur/gimeracil/oteracil potassium; SD, stable disease; VATS, video-assisted thoracic surgery.

### 3.2 Residual nodal metastases show heterogeneous morphology and squamous differentiation

Histological examination confirmed pCR in both the primary biliary lesion and the resected pulmonary metastasis. The lung lesion showed fibrosis with preserved alveolar architecture, whereas the biliary site exhibited epithelial loss, inflammatory cell infiltration, and fibrosis without viable tumor cells. In contrast, viable carcinoma persisted in 3 of 22 regional lymph nodes.

All three metastatic lymph nodes showed prominent keratinizing squamous differentiation with p40 expression and loss of CK7 expression. One metastatic lymph node additionally showed histiocytic/macrophage-rich areas with partial spatial overlap between CEA-positive and CD68-positive staining. These findings demonstrated shared squamous differentiation together with additional inter-nodal histopathological heterogeneity. The lesion selected for detailed spatial analysis showed diffuse p40 expression and loss of CK7 expression, representing a phenotype distinct from the original adenocarcinoma (**Figure 2**).

**Figure 2:**
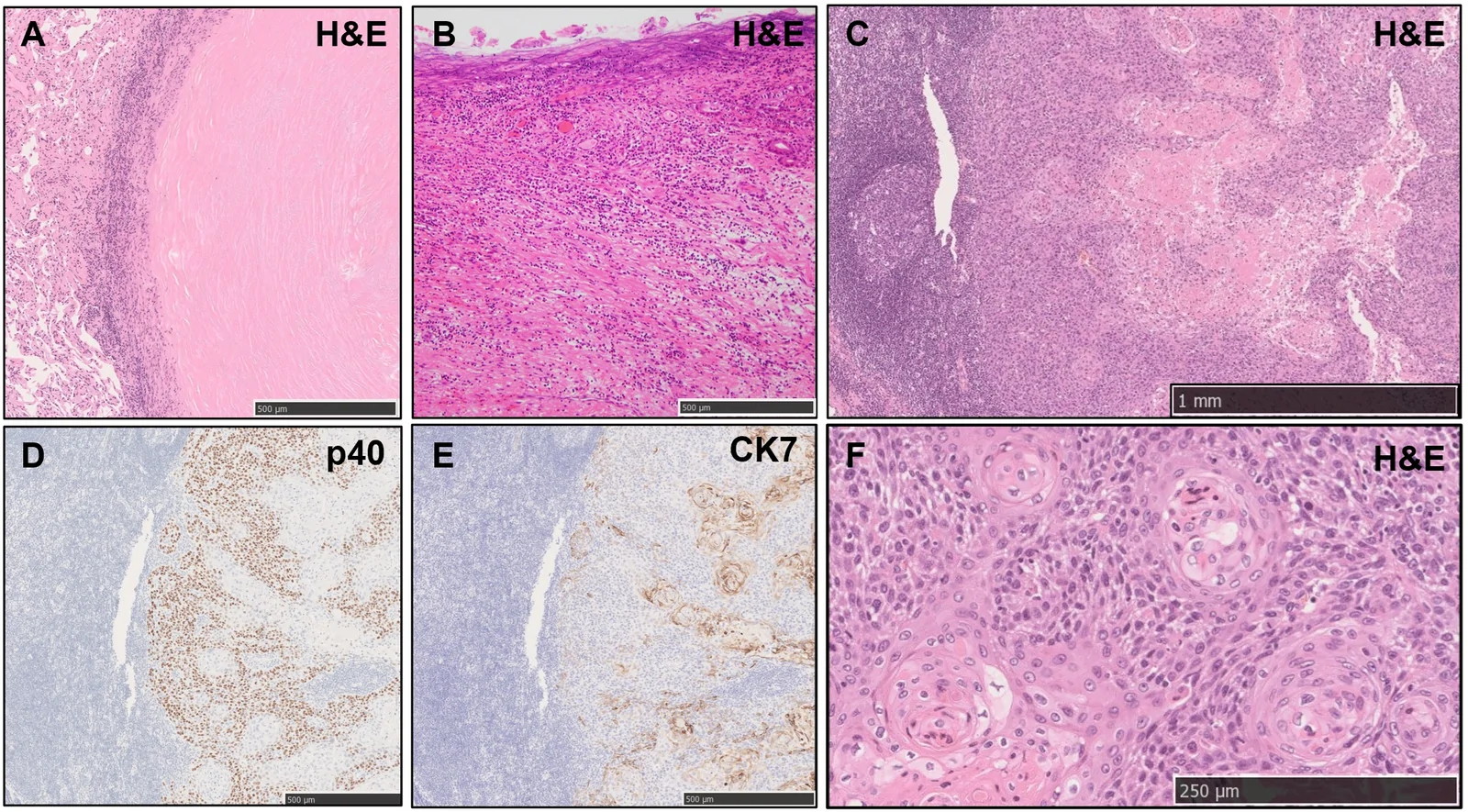
Pathological response and squamous differentiation in residual nodal metastasis. **(A, B)** Representative hematoxylin and eosin (H&E) images showing pathological complete response (pCR; Evans Grade IV) in the pulmonary metastasis **(A)** and the primary biliary tumor site **(B)**. Both sites show dense stromal fibrosis and infiltration of foamy macrophages without identifiable viable tumor cells. **(C, F)** Residual carcinoma within a regional lymph node. A low-magnification H&E image **(C)** shows tumor nests within the nodal tissue. A high-magnification view of the indicated area **(F)** shows prominent squamous differentiation with keratinization and keratin pearl formation. **(D, E)** Immunohistochemical characterization of the residual nodal tumor. Tumor cells were diffusely positive for p40 **(D)** and negative for cytokeratin 7 (CK7) **(E)**, demonstrating a squamous phenotype distinct from the original biliary adenocarcinoma. Scale bars: 500 μm **(A, B, D, E)**; 1 mm **(C)**; 250 μm **(F)**. CK7, cytokeratin 7; H&E, hematoxylin and eosin; pCR, pathological complete response.

### 3.3 Spatial immune profiling shows differential CD8 and FoxP3 distributions

Detailed spatial immune profiling was performed in one metastatic lymph node showing keratinizing squamous differentiation. CD8-positive cells accounted for a higher proportion of detected cells at the tumor margin than in the tumor core (52.7% vs. 23.3%, P = .008). To assess the potential influence of differences in cellular composition between compartments, CD8-positive cell density was additionally calculated. CD8-positive cell density was also higher at the tumor margin than in the tumor core (7,753.5 ± 2,460.4 vs. 1,925.3 ± 833.0 cells/mm², P = .008). FoxP3-positive cells also showed a higher positive-cell proportion at the margin than in the core (10.8% vs. 6.4%, P = .032). Thus, both the positive-cell proportion and cell-density analyses showed a margin-predominant distribution of CD8-positive cells within the analyzed lesion (**Figure 3**).

**Figure 3:**
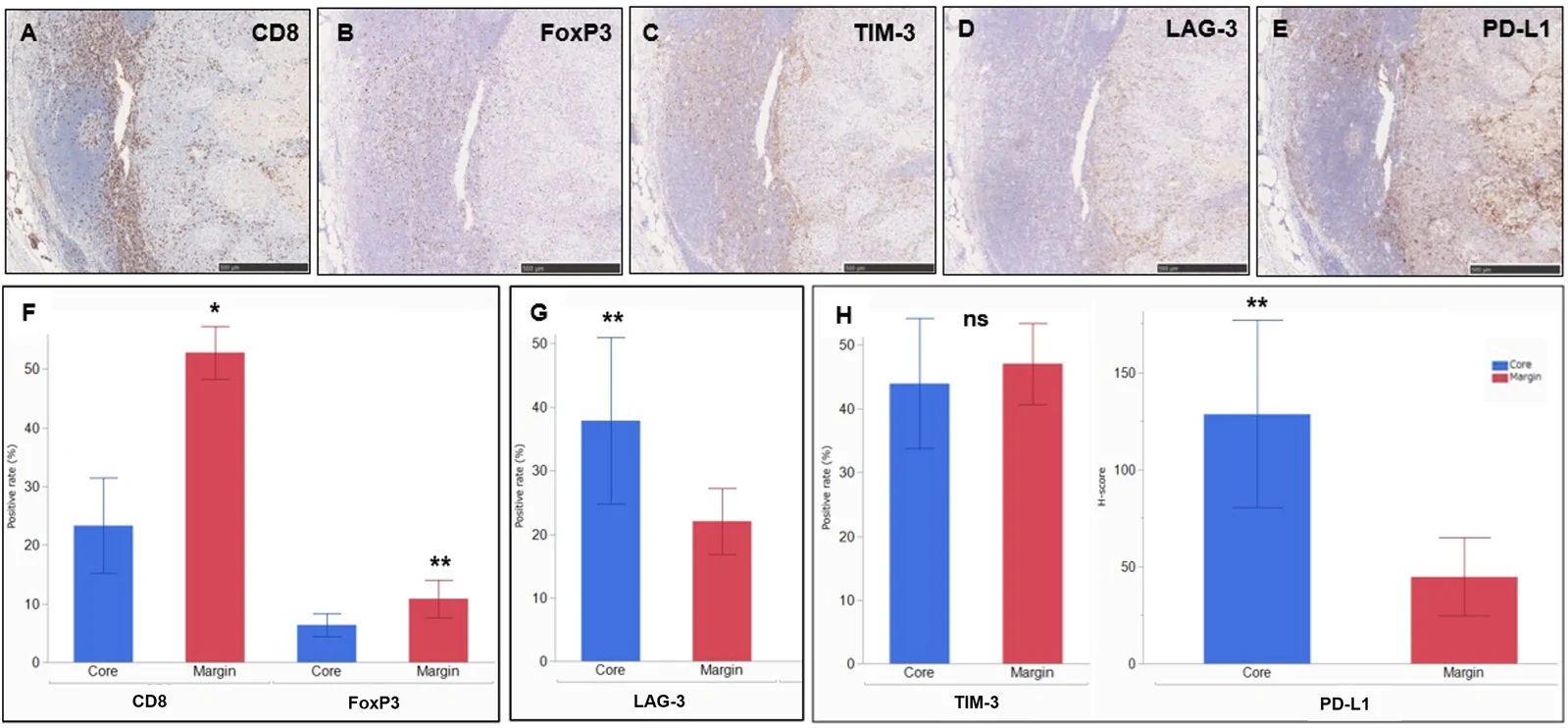
Spatial immune features of the metastatic lymph node selected for detailed quantitative analysis after chemoimmunotherapy. **(A–E)** Representative immunohistochemical images showing the spatial distributions of CD8 **(A)**, FoxP3 **(B)**, TIM-3 **(C)**, LAG-3 **(D)**, and PD-L1 **(E)** in the metastatic lymph node selected for detailed quantitative analysis. CD8-positive and FoxP3-positive cells showed higher values at the tumor margin than in the tumor core. TIM-3 expression was broadly distributed across the lesion without an obvious compartment-specific predominance, whereas LAG-3 expression and PD-L1 H-score were higher in the tumor core than at the margin. **(F–H)** Quantitative analysis of the observed spatial immune features. CD8-positive cells showed a margin-predominant distribution based on both positive-cell proportion and positive-cell density **(F)**. FoxP3-positive cells also showed a higher positive-cell proportion at the tumor margin than in the tumor core **(F)**. LAG-3 expression was higher in the tumor core than at the margin **(G)**. TIM-3 expression showed no significant difference between the tumor margin and tumor core, whereas the PD-L1 H-score was higher in the tumor core than at the margin **(H)**. The tumor margin was operationally defined as the region within 50 μm of the tumor–stroma interface, and the tumor core was defined as internal viable tumor nests, including keratinized squamous areas. Quantitative analysis was performed using QuPath within the entire manually annotated tumor margin and tumor core regions. Data are presented as mean ± SD. Statistical comparisons between the tumor margin and tumor core were performed using the Wilcoxon rank-sum test. These comparisons were exploratory within-lesion analyses and should be interpreted descriptively. *P < 0.05; **P < 0.01; ns, not significant. Scale bars, 500 μm. CD8, cluster of differentiation 8; FoxP3, forkhead box P3; H-score, histoscore; LAG-3, lymphocyte-activation gene 3; PD-L1, programmed death-ligand 1; SD, standard deviation; TIM-3, T-cell immunoglobulin and mucin-domain containing-3.

Additional CD8 and PD-L1 staining of another metastatic lymph node showed a different spatial pattern. CD8-positive cells were relatively abundant within the follicular and perifollicular lymphoid compartments but were sparse within areas showing partial spatial overlap of CEA and CD68 staining and the surrounding p40-positive tumor regions. PD-L1-positive cells were present within the lymph node but showed no preferential increase in these tumor-associated regions (**Supplementary Figure S2**).

### 3.4 Checkpoint-marker expression differs between the tumor margin and core

In the metastatic lymph node selected for detailed quantitative analysis, TIM-3 expression was high in both the tumor margin and tumor core, without a marked spatial difference. In contrast, LAG-3 expression was higher in the tumor core than at the margin (37.9% vs. 22.0%, P = .016). The PD-L1 H-score was also higher in the tumor core than at the margin (128.6 vs. 44.7, P = .032). These findings showed differential spatial distributions of checkpoint markers between the predefined tumor margin and tumor core within the analyzed nodal lesion (**Figure 3**).

## 4 Discussion

This study identifies a distinct pattern of site-specific pathological response in BTC, in which viable tumor persisted in regional lymph nodes despite pCR in the primary biliary lesion and pulmonary metastases after sequential chemoimmunotherapy. This discordant response shows that pathological treatment effects differed across disease sites, although the mechanisms underlying persistent nodal disease cannot be determined from this single case. The treatment sequence also complicates attribution of the observed pathological responses to a specific therapeutic component. Cisplatin was discontinued because of an allergic reaction rather than disease progression, and treatment was subsequently modified to one cycle of gemcitabine plus durvalumab, followed by one cycle of gemcitabine alone and durvalumab maintenance monotherapy. Therefore, the relative contributions of chemotherapy, immunotherapy, and treatment sequencing to the site-specific pathological responses cannot be determined from this single case.

A notable feature of the residual nodal disease was prominent keratinizing squamous differentiation across all three metastatic lymph nodes, accompanied by p40 expression and loss of CK7 expression. These findings show a distinct post-treatment phenotype in the residual nodal metastases. However, the origin of this phenotype cannot be determined from the present analysis. Because paired pretreatment tissue from the same nodal lesions and molecular or clonality analyses were unavailable, treatment-induced phenotypic change cannot be distinguished from selection of a pre-existing tumor subpopulation. Pre-existing intratumoral heterogeneity or an unrecognized squamous component therefore remains an alternative explanation.

One metastatic lymph node additionally showed histiocytic/macrophage-rich areas with partial spatial overlap between CEA-positive and CD68-positive staining. This finding is descriptive and does not establish a specific macrophage-mediated mechanism. Structures morphologically resembling peribiliary glands (PBGs) were also observed. PBGs have been reported to contain biliary stem/progenitor cell populations (15). However, the present observation was based solely on morphology and does not establish a progenitor niche or a mechanistic relationship with the squamous phenotype. Thus, the present findings document post-treatment morphological and immunophenotypic heterogeneity but do not establish lineage plasticity as a mechanism of treatment resistance.

Spatial immune profiling showed different distributions of immune markers between the predefined tumor margin and tumor core in the metastatic lymph node selected for detailed quantitative analysis. CD8-positive cells showed a margin-predominant distribution based on both positive-cell proportion and cell density, whereas FoxP3-positive cells also showed a higher positive-cell proportion at the margin. These findings describe spatial heterogeneity within the analyzed lesion but do not establish a structural mechanism responsible for the observed distribution.

Additional evaluation of another metastatic lymph node demonstrated a different CD8 distribution, with relatively abundant CD8-positive cells in the follicular and perifollicular lymphoid compartments but fewer CD8-positive cells within tumor-associated regions. This inter-nodal difference indicates that the spatial immune pattern observed in the quantitatively analyzed lesion should not be generalized to all metastatic lymph nodes in this patient.

Because FoxP3 single staining does not define the functional state of these cells, the observed FoxP3 distribution should be interpreted descriptively rather than as evidence of functional immune suppression (16). LAG-3 expression and the PD-L1 H-score were higher in the tumor core than at the margin in the quantitatively analyzed lesion, whereas TIM-3 expression was broadly distributed across both compartments. Because these analyses did not identify the specific cell populations expressing PD-L1, LAG-3, or TIM-3, the observed spatial distributions do not establish the cell type-specific or functional significance of these markers.

Together, these findings are summarized schematically for the metastatic lymph node selected for detailed quantitative analysis (**Supplementary Figure S1**). This schematic represents the spatial immune features observed in that lesion and is not intended to represent all metastatic lymph nodes in this patient. Additional evaluation of another metastatic lymph node showed a different CD8 distribution, further indicating inter-nodal heterogeneity in the local immune landscape. These observations are consistent with previous spatial and single-cell studies demonstrating immune heterogeneity across anatomical compartments in cholangiocarcinoma (7–10). However, the present analysis did not directly evaluate structural components of the lymph node, such as high endothelial venules, lymphatic sinuses, or fibrotic barriers. Therefore, the observed spatial distributions do not establish an anatomical mechanism of immune exclusion.

From a clinical perspective, discordant responses across disease sites remain an important challenge in patients receiving immunotherapy (2). Regional lymphadenectomy is routinely performed as part of curative-intent resection for perihilar cholangiocarcinoma, and recent data support evaluation of at least six regional lymph nodes (17). Although 22 regional lymph nodes were examined in the present case, the frequency of this specific pattern of nodal persistence after chemoimmunotherapy remains unknown. In this case, pathological confirmation of pCR in the pulmonary lesion supported the decision to proceed with radical surgery. Complete resection was achieved despite marked fibrosis. This clinical course suggests that surgical consolidation may warrant consideration in carefully selected patients who show a favorable systemic response but retain potentially resectable residual disease.

This study has several limitations. First, it represents a single-case analysis, and the findings cannot be generalized without further validation. Because cisplatin was discontinued after an allergic reaction and treatment was subsequently modified, the relative contributions of chemotherapy, immunotherapy, and treatment sequencing to the observed pathological responses cannot be determined. Detailed quantitative spatial immune analysis was performed in one metastatic lymph node, whereas additional CD8 and PD-L1 staining of another metastatic lymph node was evaluated descriptively. The additional lesion showed a different CD8 distribution, indicating inter-nodal heterogeneity and further limiting generalization of the spatial pattern observed in the quantitatively analyzed lesion. The five regional measurements within each compartment of the quantitatively analyzed lymph node were not independent biological replicates. Limited regional sampling and multiple marker comparisons also increase the potential for chance findings; therefore, the reported P values should be interpreted descriptively and with caution. The predefined 50-μm boundary used to define the tumor margin was an operational spatial threshold and may have influenced the observed differences between the tumor margin and tumor core. In addition, structural components of the lymph node, including high endothelial venules, lymphatic sinuses, and fibrotic barriers, were not specifically evaluated. Accordingly, the spatial comparisons should be interpreted as descriptive within-lesion observations rather than evidence of a generalizable mechanism of immune exclusion.

Second, the morphological and immunophenotypic findings do not establish therapy-induced lineage plasticity. Paired pretreatment tissue from the same nodal lesions and molecular or clonality analyses were not available. Therefore, treatment-induced phenotypic change cannot be distinguished from selection of a pre-existing tumor subpopulation, and neither pre-existing intratumoral heterogeneity nor an unrecognized squamous component can be excluded.

Third, the immune microenvironment was characterized using a limited panel of immune markers. Although CD8, FoxP3, PD-L1, LAG-3, and TIM-3 provided an initial description of the spatial immune landscape, CD4-positive T cells, the functional state of CD8-positive T cells (e.g., Granzyme B, Ki67, and PD-1), antigen-presenting cells, B cells, and tertiary lymphoid structures were not systematically evaluated. CD68 staining was additionally performed in one metastatic lymph node for pathological characterization of histiocytic/macrophage-rich areas, but macrophages were not included in the comparative spatial immune profiling. Furthermore, FoxP3 single staining cannot distinguish activated effector regulatory T cells from resting regulatory T cells (16). PD-L1 was evaluated using a regional H-score within the annotated regions, and separate tumor-cell and immune-cell scoring (TPS and IC) was not performed. The specific cell populations expressing LAG-3 and TIM-3 were also not identified. Therefore, the cellular sources and cell type-specific biological significance of these checkpoint-marker signals remain unresolved. In addition, comparison with non-tumor-bearing lymph nodes was not performed; therefore, the observed marker distributions should be interpreted as relative spatial differences within the analyzed metastatic lesions rather than as deviations from normal lymph-node expression.

Despite these limitations, the combined pathological and spatial findings show persistent nodal disease characterized by prominent keratinizing squamous differentiation across all three metastatic lymph nodes and inter-nodal heterogeneity in immune-cell distribution after chemoimmunotherapy in BTC. Further studies incorporating paired pretreatment specimens, molecular or clonality analyses, and broader comparative spatial immune profiling across multiple metastatic lesions are needed to determine the biological basis and generalizability of these observations.

## Data Availability

The data are not publicly available because they contain information that could compromise the privacy of the research participant. The data that support the findings of this study are available from the corresponding author upon reasonable request.

## Supplementary material

Supplementary Figure 1 Schematic summary of spatial immune findings in the metastatic lymph node selected for detailed quantitative analysis Schematic representation of the spatial immune findings observed in the metastatic lymph node selected for detailed quantitative analysis. This figure summarizes findings from that representative keratinizing lesion and is not intended to represent all metastatic lymph nodes in this patient. The outer region corresponds to the predefined tumor margin, where CD8-positive and FoxP3-positive cells showed margin-predominant distributions. The intermediate region represents the broad distribution of TIM-3 expression across the lesion without a marked spatial difference between the tumor margin and tumor core. The inner region corresponds to the tumor core, where LAG-3 expression and PD-L1 H-score were higher than at the tumor margin. The accompanying table summarizes the principal spatial immune findings observed in this lesion. This schematic is intended only as a descriptive summary of the quantitatively analyzed metastatic lymph node and does not imply that the same spatial pattern was present in the other metastatic lymph nodes or establish a specific biological mechanism.

Supplementary Figure 2 Additional pathological and immune characterization of another metastatic lymph node. Representative serial sections from another metastatic lymph node showing prominent keratinizing squamous differentiation. **(A)** Hematoxylin and eosin staining. **(B)** p40 immunohistochemistry showing p40-positive tumor regions. **(C)** Cytokeratin 7 (CK7) staining showing loss of CK7 expression in the tumor. **(D, E)** CEA and CD68 staining showing partial spatial overlap between CEA-positive and CD68-positive areas. **(F)** CD8-positive cells were relatively abundant in the follicular and perifollicular lymphoid compartments but were sparse within areas showing partial overlap of CEA and CD68 staining and the surrounding p40-positive tumor regions. **(G)** PD-L1-positive cells were present within the lymph node but showed no preferential increase in these tumor-associated regions. These findings were assessed descriptively and were not included in the quantitative margin-versus-core analysis performed in the metastatic lymph node shown in **Figure 3**. CEA, carcinoembryonic antigen; H&E, hematoxylin and eosin; PD-L1, programmed death-ligand 1.
