# Supplementary material for: Persistent nodal disease with squamous differentiation and spatial immune heterogeneity after chemoimmunotherapy in biliary tract cancer: a high-resolution pathological analysis
Source: Front Immunol. 2026 Oct 23;17:1855354. doi: 10.3389/fimmu.2026.1855354 (PMC13739777; doi:10.3389/fimmu.2026.1855354)
Supplement: Supplementary file 1 [file Table1.docx]

**Supplementary Table S1. Summary of annotated regions and analyzed cell numbers used for quantitative spatial immune analysis.**

| Marker | Tumor margin (mm²) | Tumor core (mm²) | Total annotated area (mm²) | Total analyzed cells* |
| --- | --- | --- | --- | --- |
| CD8 | 3.84 | 5.43 | 9.27 | 67955 |
| FoxP3 | 3.99 | 6.04 | 10.03 | 84499 |
| PD-L1 | 3.79 | 7.50 | 11.29 | 126793 |
| LAG-3 | 3.13 | 4.86 | 7.99 | 174130 |
| TIM-3 | 4.12 | 5.46 | 9.58 | 129225 |

* Total analyzed cells represent the total number of cells detected by QuPath within the combined annotated tumor margin and tumor core regions for each immunohistochemical marker. Quantitative measurements were performed within the entire annotated regions rather than on representative microscopic fields.
