# Supplementary material for: Persistent nodal disease with squamous differentiation and spatial immune heterogeneity after chemoimmunotherapy in biliary tract cancer: a high-resolution pathological analysis
Source: Front Immunol. 2026 Oct 23;17:1855354. doi: 10.3389/fimmu.2026.1855354 (PMC13739777; doi:10.3389/fimmu.2026.1855354)
Supplement: Supplementary file 2 [file Table2.docx]

**Supplementary Table S2. QuPath cell detection and marker-specific classification parameters used for digital spatial immune analysis.**

| **Parameter** | **CD8, FoxP3, LAG-3, TIM-3** | **PD-L1** |
| --- | --- | --- |
| Detection image | Hematoxylin OD | Hematoxylin OD |
| Requested pixel size (μm) | 0.5 | 0.5 |
| Background radius (μm) | 8 | 10 |
| Sigma (μm) | 1.5 | 1.5 |
| Median filter radius (μm) | 0 | 1 |
| Minimum nucleus area (μm²) | 10 | 25 |
| Maximum nucleus area (μm²) | 400 | 400 |
| Detection threshold | 0.10 | 0.05 |
| Cell expansion (μm) | 1 | 3 |
| Score compartment | Nucleus: DAB OD mean | Nucleus: DAB OD mean |
| Positive classification | Single-threshold DAB optical density classification | Three-level DAB optical density classification (H-score) |
| Positive threshold | 0.10 | - |
| Threshold 1+ | N/A | 0.12 |
| Threshold 2+ | N/A | 0.20 |
| Threshold 3+ | N/A | 0.28 |

All nucleated cells were automatically detected using the hematoxylin optical density channel within the annotated regions. CD8, FoxP3, LAG-3, and TIM-3 were classified as positive or negative using a single DAB optical density threshold. PD-L1 staining intensity was classified using three thresholds (1+, 2+, and 3+) for calculation of the H-score. Cell detection and marker classification results were visually reviewed for concordance with the corresponding immunohistochemical staining patterns.

**Abbreviations:** DAB, 3,3′-diaminobenzidine; OD, optical density; PD-L1, programmed death-ligand 1; N/A, not applicable
